# Availability of Amino Acids Extends Chronological Lifespan by Suppressing Hyper-Acidification of the Environment in *Saccharomyces cerevisiae*

**DOI:** 10.1371/journal.pone.0151894

**Published:** 2016-03-18

**Authors:** Yo Maruyama, Toshiyuki Ito, Hiroaki Kodama, Akira Matsuura

**Affiliations:** 1 Department of Nanobiology, Graduate School of Advanced Integration Science, Chiba University, Inage-ku, Chiba, 263–8522, Japan; 2 Molecular Chirality Research Center, Chiba University, Inage-ku, Chiba, 263–8522, Japan; 3 Keiyo Plant Engineering Co., Ltd., 2-8-8 Ichikawaminami, Ichikawa, Chiba, 272–0032, Japan; University of Tokyo, JAPAN

## Abstract

The chronological lifespan of *Saccharomyces cerevisiae* represents the duration of cell survival in the postdiauxic and stationary phases. Using a prototrophic strain derived from the standard auxotrophic laboratory strain BY4742, we showed that supplementation of non-essential amino acids to a synthetic defined (SD) medium increases maximal cell growth and extends the chronological lifespan. The positive effects of amino acids can be reproduced by modulating the medium pH, indicating that amino acids contribute to chronological longevity in a cell-extrinsic manner by alleviating medium acidification. In addition, we showed that the amino acid-mediated effects on extension of chronological longevity are independent of those achieved through a reduction in the TORC1 pathway, which is mediated in a cell-intrinsic manner. Since previous studies showed that extracellular acidification causes mitochondrial dysfunction and leads to cell death, our results provide a path to premature chronological aging caused by differences in available nitrogen sources. Moreover, acidification of culture medium is generally associated with culture duration and cell density; thus, further studies are required on cell physiology of auxotrophic yeast strains during the stationary phase because an insufficient supply of essential amino acids may cause alterations in environmental conditions.

## Introduction

All living organisms are subjected to physiological decline with age, which restricts the lifespan of the organism. Recent advances by studies on the mechanisms of aging have revealed many causal factors, including oxidative stress, telomere shortening, mitochondrial dysfunction, epigenetic changes and loss of protein quality control. However, the primary causes of aging, if any, remain controversial.

The budding yeast *Saccharomyces cerevisiae* (*S*. *cerevisiae*) has been used as a model organism to study the mechanism of aging at the cellular level using powerful genetic and post-genomic approaches. Two types of aging are known in this organism, replicative aging and chronological aging. Due to the asymmetric cell division of this organism, damaged cellular materials segregate unequally in each division, causing accumulation of damaged materials in a mother cell. Thus, an individual mother cell ages after successive replication, ceases cell division and undergoes cell death.

*S*. *cerevisiae* cells grown in liquid medium continue to proliferate until carbon sources are exhausted and the culture enters the stationary phase. The chronological lifespan of *S*. *cerevisiae* represents the duration of cell survival in the postdiauxic and stationary phases and has been used as a model for the senescence processes of postmitotic tissues or cells in higher eukaryotes. During chronological aging of yeast, limited nutrient availability leads to programmed cell death of unfit cells, which appears to be beneficial for the fitter ones in the population [1]. Several causes of cell death have been suggested, such as mitochondrial dysfunction [2–4], accumulation of reactive oxygen species [5–7], reduction of protein quality control [8], replication stress due to cell cycle arrest outside G1 [9] and decline of cellular ATP levels [10]. However, the exact mechanism, including the interrelationships of these phenomena, remains unclear.

The nutrient environment is an important factor of aging. Chronological aging is regulated by the two nutrient-sensing signaling pathways involving Ras-PKA and TORC1. Genetic manipulation or interventions directing the downregulation of these pathways, such as caloric restriction or the addition of the TORC1 inhibitor rapamycin, extends the chronological lifespan. The action of the two pathways in chronological lifespan is mediated by protein kinase Rim15p, which promotes the activation of Msn2/4p and Gis1p, which are involved in stress-responsive transcription [11].

Caloric restriction is usually imposed by a reduction in the glucose content of the medium in yeast experiments. In addition, a progressive reduction of non-essential amino acid concentrations promotes replicative lifespan extension in yeast [12]. In contrast, auxotrophic mutants show a reduced chronological lifespan under an insufficient supply of essential amino acids, suggesting that the positive effect of caloric restriction on lifespan is restricted to non-essential nutrients [13].

During liquid culturing of *S*. *cerevisiae*, the medium pH decreases due to secreted metabolites. Based on previous studies, a model was proposed stating that extrinsic acetic acid drives medium acidification and is the primary cause of chronological aging in yeast [6, 14–16]. Others argued that acetic acid and media acidification are separate and non-exclusive pro-aging factors [1, 17]. In the present study, using a prototrophic derivative of the standard *S*. *cerevisiae* strain BY4742, we demonstrated that acidification, but not acetic acid concentration, limits chronological lifespan and maximal cell density in a liquid synthetic medium. Availability of non-essential amino acids as a nitrogen source alleviates acidification, while the uptake of ammonium ions prior to the diauxic shift leads to hyper-acidification and harmful effects on growth and viability of yeast cells.

## Materials and Methods

### Strains and growth conditions

YOM36, a prototrophic derivative of BY4742 (*MAT*α *his3*Δ*1 leu2*Δ*0 lys2*Δ*0 ura3*Δ*0*), was constructed by sequential integration of the corresponding wild-type genes. To achieve this, *URA3* and *LYS2* fragments were amplified using genomic DNA of X2180-1A and BY4741, respectively, and those for *HIS3* and *LEU2* were isolated from plasmids pJJ215 and pJJ283 [18], respectively. Σ1278b (also known as MB1000)[19] was obtained from H. Takagi.

Chronological lifespan assays were performed as described previously [20]. All cultures were initiated by seeding a single colony on SD medium into 5 mL of SD medium overnight and a portion of the culture was inoculated into SD or SC medium, adjusting the initial OD_600_ value to 0.01. Cultures in flasks were grown at 30°C with shaking at 200 rpm. Growth was monitored by measuring OD_600_ and viability was determined by counting colony-forming units (CFUs) every 2 days of incubation on YPD agar plates. The CFUs on day 3 were considered to denote 100% survival. SD medium was composed of 0.17% yeast nitrogen base without amino acids and ammonium sulfate (BD Difco), 0.5% ammonium sulfate and 0.2% glucose and SC medium was prepared by supplementing amino acids and nucleobases to SD medium at the concentrations described previously [21], namely 20 mg/L each of adenine, uracil, tryptophan, histidine, arginine, and methionine, 30 mg/L each of tyrosine, isoleucine, and lysine, 50 mg/L of phenylalanine, 60 mg/L of leucine, 100 mg/L each of glutamic acid and aspartic acid, 150 mg/L of valine, 200 mg/L of threonine, and 400 mg/L of serine.

### Media swap experiments

At day 3, cultures were subjected to centrifugation at 3,000 rpm for 3 min. The supernatant was filtrated through 0.45 μm pore-sized membranes (Sartorius) and the filtrated medium was combined with the cell pellet from another culture.

### Capillary electrophoresis analysis

Capillary electrophoresis was performed using a G1600A system (Agilent Technologies) as previously described [22]. Culture medium was pretreated with an ultrafiltration membrane (cutoff <3,000 kNMWL) and separations were performed on a fused silica capillary (G1600-64211; Agilent) with 50 μm id, L = 112.5 cm (104 cm effective length). Samples were injected at a pressure of 50 mbar for 6.0 s. Detection was performed with an indirect photometric method using wavelengths of 350 nm and 230 nm. The applied voltage was set at –30 kV and the capillary temperature was 15°C.

### Measurement and adjustment of medium pH

Medium pH was measured using a pH electrode (Mettler Toledo). To adjust the pH of the culture medium, cells were separated by centrifugation and the pH medium was adjusted with 1 N NaOH or 1 N HCl. The pH-adjusted medium was recombined with the cells after sterile filtration using a 0.45 μm pore-sized membrane (Sartorius).

### Measurement of glucose concentrations

Glucose levels in media were measured using the spectrophotometric method with a Glucose CII Test kit (Wako).

### Reproducibility of the results

The results presented are mean values of at least three independent assays, and data are presented as means ± standard deviations. Statistical evaluations were carried out using two-way ANOVA.

## Results

### Amino acids extend chronological lifespan of prototrophic yeast

Although in a natural habitat *S*. *cerevisiae* strains can assimilate inorganic nitrogen sources into all types of vital amino acids, experimentally used strains are usually auxotrophic and, thus, deficient in the production of specific amino acids. Previous studies showed that concentrations of auxotrophic amino acids affect the chronological lifespan [13], indicating that supply of essential nutrients affects longevity.

**Fig 1:**
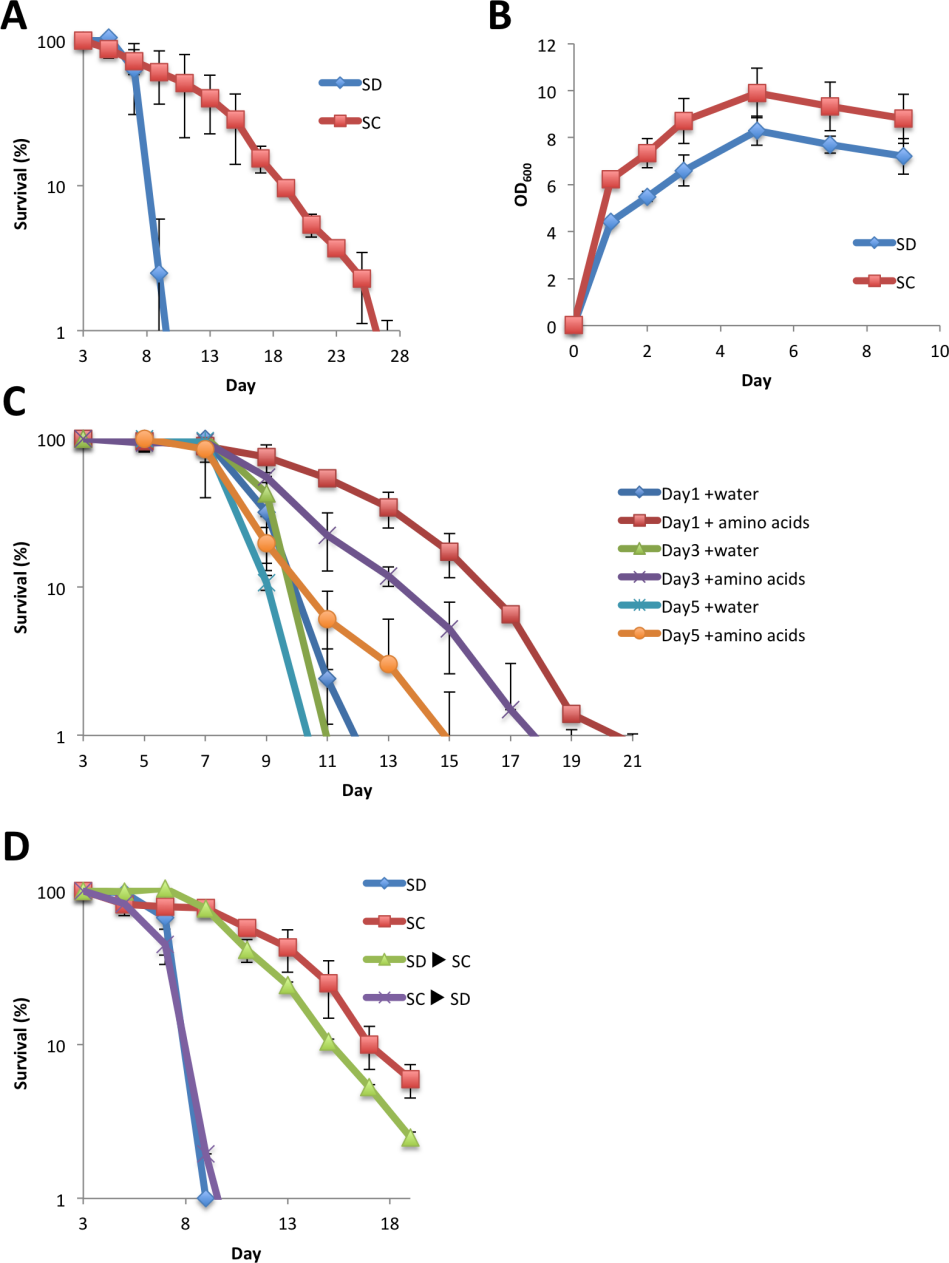
Addition of non-essential amino acids extended the chronological lifespan of a prototrophic yeast strain. (A), (B) Chronological lifespan and cell growth in a synthetic medium with or without amino acids. YOM36 cells grown in SD medium were transferred to SD or SC media and CFUs and cell density of the cultures were measured at the indicated times. (C) Effect of late addition of amino acids. Amino acid mixture or water was added to cultures of YOM36 cells grown in SD for the indicated times and chronological lifespan assay was performed as in (A). (D) Effect of media swap on chronological lifespan. The original media of the cells grown as in (A) and (B) were swapped at day 3. Error bars represent the means ± SD (n = 3). (A) *P*<0.01; (B) *P*<0.01; (C) *P*<0.01 (Day 1 water *vs* Day 1 amino acids; Day3 water *vs* Day 3 amino acids), *P*<0.05 (Day 5 water *vs* Day 5 amino acids); (D) *P*<0.01 (SD *vs* SD→SC; SC *vs* SC→SD).

In the chronological aging experiments, synthetic complete (SC) medium, an amino acid-supplemented medium, is commonly used. SC medium is based on synthetic defined (SD) medium composed of yeast nitrogen base, ammonium sulfate and glucose, with 14 amino acids and two nucleobases (adenine and uracil) supplemented at the concentrations described previously [21]. To address the effect of non-essential amino acid addition on yeast growth and chronological longevity, we constructed a prototrophic strain, YOM36, from the standard strain, BY4742, by introducing the corresponding wild-type alleles of auxotrophic mutations at the original genomic loci. As shown in Fig 1A and 1B, the presence of amino acids in the medium still impacted the chronological lifespan of YOM36; cells in SC medium showed higher cell concentrations in the stationary phase and maintained viability for a longer period than in SD. This result shows that the previously reported effects of extracellular amino acids on survival of yeast cells are unrelated to auxotrophy of experimental strains.

Despite the differences in final cell concentrations, the growth phase of the culture proceeded similarly in SD and SC, meaning cells at days 1, 3 and 5 corresponded to before and after the diauxic shift and stationary phase, respectively, in both media (Fig 1B). Next, we examined the timing of amino acid action in extending the chronological lifespan using a delayed administration experiment. YOM36 cells were cultivated in SD and amino acids were added to the medium at 1, 3 and 5 days after initiation of the culture. As shown in Fig 1C, addition of amino acids at any time point significantly improved the survival of stationary cells, with earlier additions being more effective. This result demonstrates that amino acid addition improves the chronological lifespan more effectively when present during the growing stages of the culture.

### Presence of amino acids affects chronological lifespan via cell-extrinsic factors

Two alternative mechanisms may explain the improvement of cellular survival by the addition of amino acids. Cells in the stationary phase acquire resistance to stress, such as heat shock and oxidation, and addition of amino acids may extend the lifespan by improving cell stress resistance. Alternatively, addition of amino acids may alter the environmental conditions directly or indirectly *via* secreted substances from the cells; yeast cells release organic acids into the medium and acetic acid released from the cells reduces the chronological lifespan [15]. To investigate whether the effect of amino acid administration on chronological lifespan is attributed to intrinsic changes in the cells or the exogenous factors present in the medium, we performed a medium-swap experiment. In this experiment, cells were initially cultured in SD or SC media and cells at day 3 were separated from the original medium by centrifugation, combined with the other medium collected from a parallel culture and further incubated to examine survival. As shown in Fig 1D, the chronological lifespan was increased when cells in SD medium were resuspended in SC medium, but decreased when the medium was switched from SC to SD medium. These results show clearly that extracellular factors present in the SC medium mediate the effect of amino acids.

**Fig 2:**
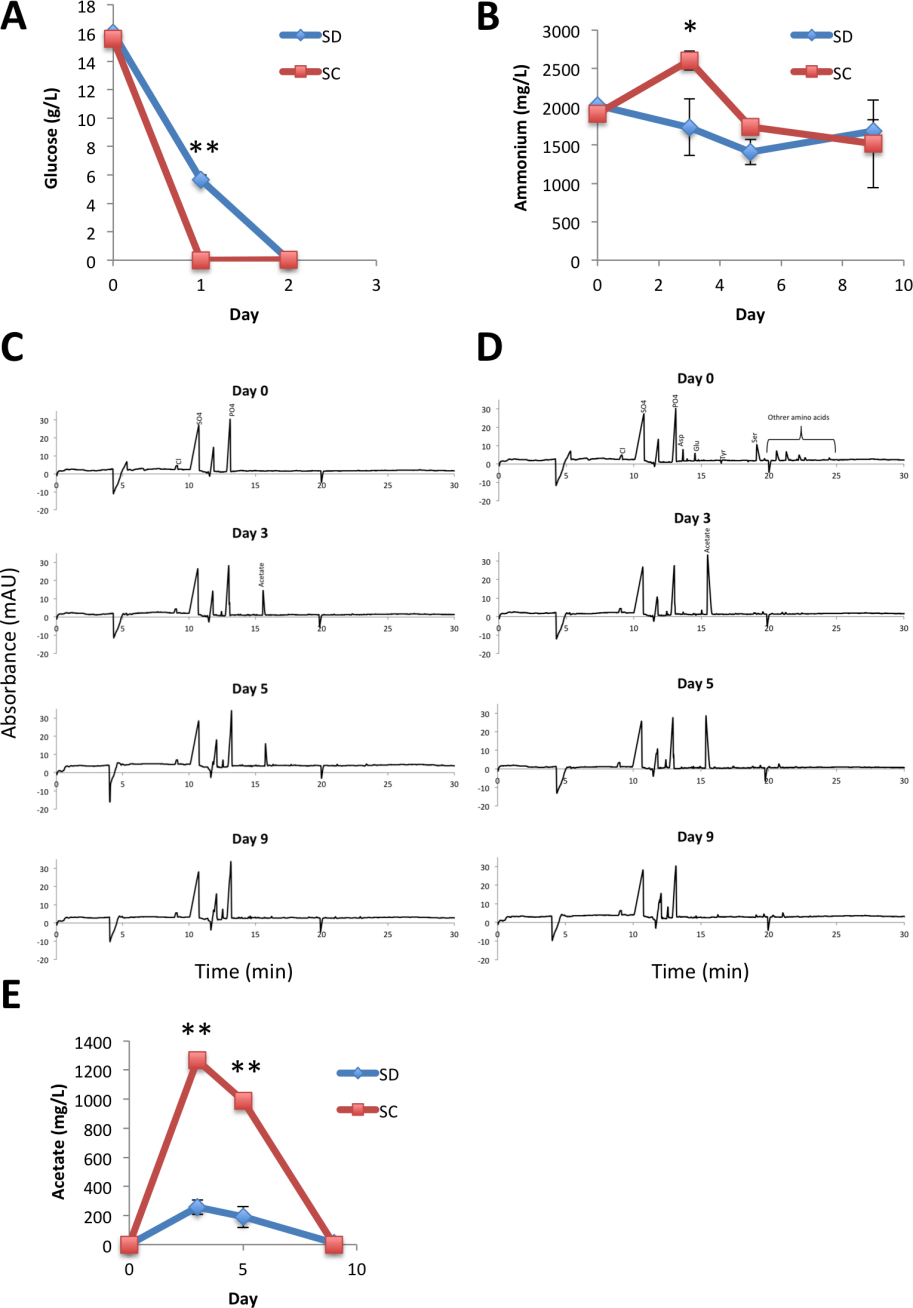
Changes in substance content of the medium during chronological aging. (A) Glucose concentration. (B) Ammonium concentration. (C), (D) Capillary electrophoresis analysis of the media. (E) Acetate concentration. Culture media of YOM36 cells grown in SD (C) or SC (D) as in Fig 1 were recovered at the indicated times and inorganic anions, organic acids and amino acids in the media were analyzed using the capillary zone electrophoresis method. Error bars represent the means ± SD (n = 3). *P<0.05; **P<0.01.

Based on the above results, we next examined the difference in medium composition of SD and SC cultures and the chronological changes. Glucose was consumed during the initial 3 days in both cultures, but the ammonium ion level was maintained at 80% of the initial concentration at day 9, indicating the nitrogen source was still available from the medium to cells at the stationary phase (Fig 2A and 2B). Capillary electrophoresis analysis showed that the amounts of nitrogen sources and acetate differed between time-matched samples of SD and SC cultures; supplemented amino acids in SC mainly disappeared at day 3, thus, the remaining amino acids apparently were not the longevity factor present in SC medium (Fig 2C and 2D). Interestingly, the amount of ammonium ions transiently increased in SC culture at day 3, indicating that cells under amino acid-rich conditions released metabolism-derived ammonium into the medium.

The production of acetic acid triggers senescence in glucose-based medium [15]. Under our experimental conditions, acetate accumulated at day 3 and disappeared at day 9 (Fig 2E). Inconsistent with previous observations that acetate accumulation enhanced chronological aging, the acetate levels were higher in SC, the medium in which cells showed a longer lifespan.

**Fig 3:**
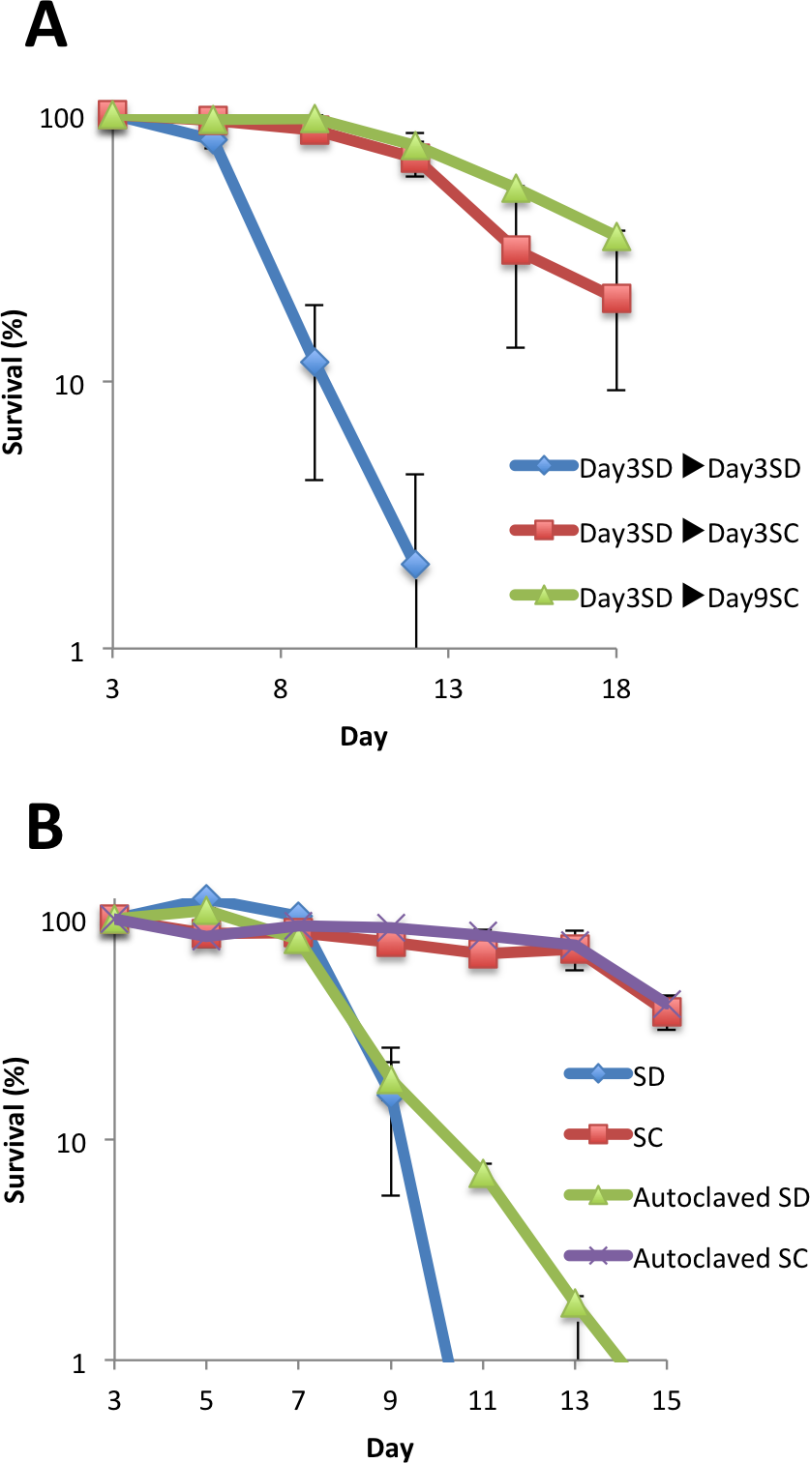
Used SC media contained a heat-resistant factor for chronological longevity. (A) Effect of media swap using older cultures. The original media of the cells grown in SD as in Fig 1 were swapped to the medium recovered from the culture in SD for 3 days, SC for 3 days, or SC for 9 days. (B) Autoclaving did not affect the longevity activity in used SC medium. Culture media of YOM36 cells at day 3 were recovered, and the supernatants were autoclaved at 121°C for 15 min. The autoclaved medium was recombined with the original, centrifuged cell pellet and then CFUs of the culture were examined at the indicated times. Error bars represent the means ± SD (n = 3). (A) *P*<0.01 (Day 3 SD→Day 3 SD *vs* Day 3 SD→Day 3 SC; Day 3 SD→Day 3 SD *vs* Day 3 SD→Day 9 SC).

To further examine the relationship between the postulated longevity factor and chemical composition of the medium, we repeated the medium-swap experiment using older cultures. When cells grown in SD medium at day 3 were combined with SC medium from day 9, the cells maintained comparable longevity to those combined with SC at day 3 (Fig 3A). Because acetate in the medium returned to non-detectable levels at day 9, acetate was not the longevity factor present in the SC medium. In addition, autoclaving the used SC medium at day 3 did not affect the longevity activity (Fig 3B), suggesting that the activity was not mediated through heat-labile molecules, such as a proteins or peptides.

### Amino acids contribute to chronological longevity by alleviating medium acidification

**Fig 4:**
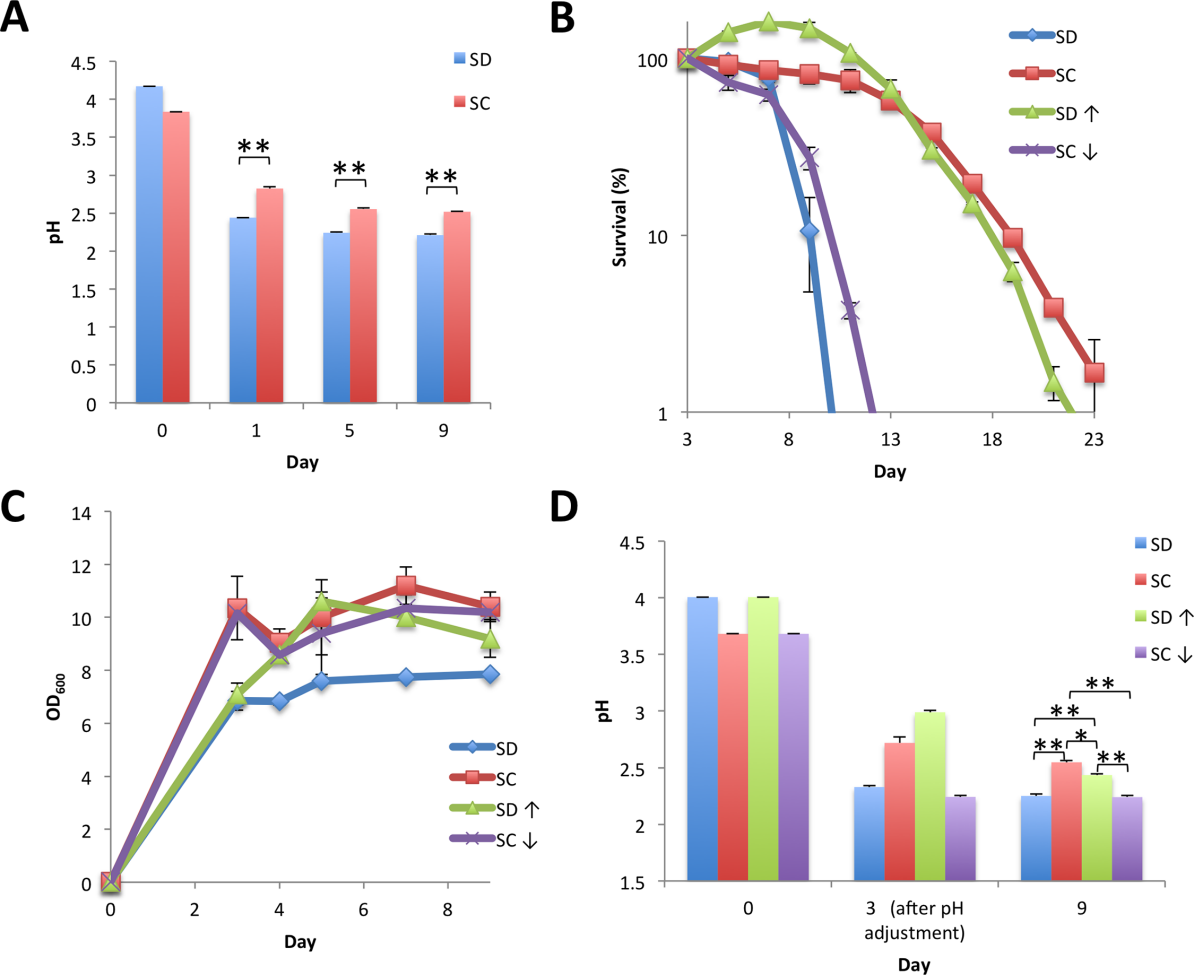
Extracellular pH is an extrinsic factor that mediates chronological aging. (A) Changes in medium pH. Medium pH of YOM36 cells grown in SD or SC as in Fig 1 were measured at the indicated times. (B), (C), (D) Chronological lifespan and cell growth after pH adjustment. Culture media of YOM36 cells at day 3 were recovered and pH of the SC culture and SD culture were adjusted to 2.2 or 3.0 with HCl or NaCl, respectively. The pH-adjusted used medium was recombined with the original, centrifuged cell pellet and then CFUs (B), cell density (C), and medium pH (D) of the culture were examined at the indicated times. Error bars represent the means ± SD (n = 3). (A) **P<0.01; (B) *P*<0.01 (SD vs SD⇑; SC vs SC⇓); (C) *P*<0.01 (SD vs SD⇑; SD vs SC⇓); (D) *P<0.05; **P<0.01.

The production of acetic acid and subsequent acidification of the medium was suggested to trigger the transition from quiescence to senescence. Measuring the medium pH revealed that pH values decreased progressively during cultivation both in SD and in SC cultures. The degree of the reduction was robust for the initial 3 days and the subsequent 6 day pH in SC remained slightly, but significantly, higher than in SD (Fig 4A).

To further examine the contribution of the difference in pH to chronological senescence, we examined the effect of pH manipulation in culturing media at day 3. To achieve this, culturing media were recovered by centrifugation and then the SD medium pH was adjusted to 3.0 by the addition of NaOH, or in SC to 2.2 by the addition of HCl. The pH-adjusted medium was combined with the corresponding cells and cultivation was resumed. As shown in Fig 4B and 4C, chronological survival in pH 3.0-adjusted SD was comparable to that in SC, whereas cells lost viability earlier in pH 2.2-adjusted SC similar to those in SD. The difference in pH between pH-treated and -untreated media was maintained at day 9 (Fig 4D). These results suggest that delay of chronological aging in amino acid-supplemented medium is due to the difference in medium pH in stationary cultures.

### Amino acid-mediated effects on extension of chronological longevity are independent of those achieved by TORC1 inhibition

**Fig 5:**
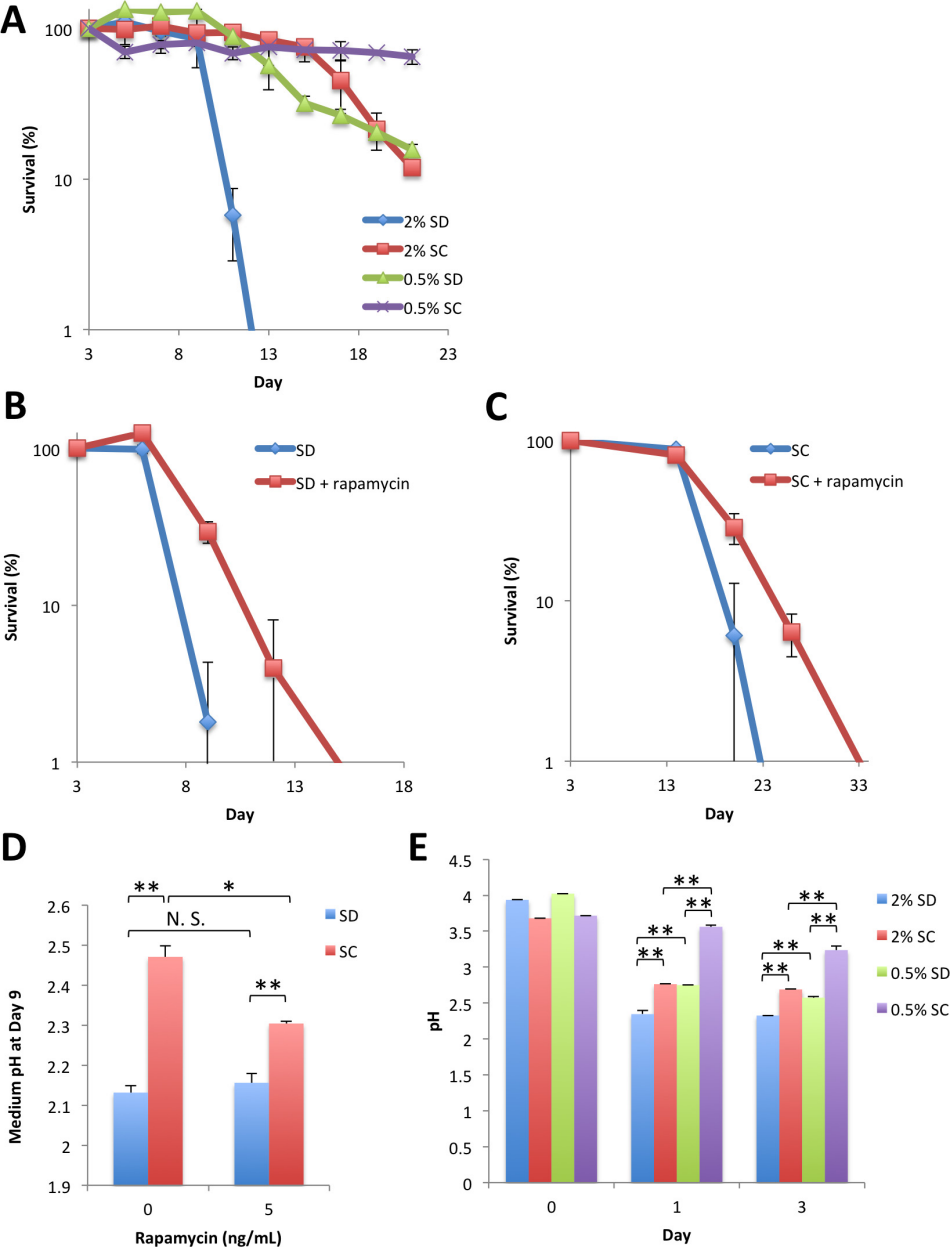
Caloric restriction- or rapamycin-mediated chronological longevity is independent of extracellular pH. (A) Effect of caloric restriction. YOM36 cells were transferred to SD or SC media containing either 2% (normal) or 0.5% (caloric-restricted) glucose. CFUs were measured at the indicated times. (B), (C) Effect of rapamycin. YOM36 cells were transferred to SD (B) or SC media (C) with or without 5 ng/mL rapamycin. CFUs were measured at the indicated times. (D) Effect of rapamycin on medium pH. Medium pH of the cultures grown as in (B) and (C) at day 9 was measured. (E) Effect of caloric restriction on medium pH. Medium pH of the cultures grown as in (A) was measured at the indicated times. Error bars represent the means ± SD (n = 3). (A) *P*<0.01 (2% SD *vs* 0.5% SD; 2% SC *vs* 0.5% SC) (B) *P*<0.01; (C) *P*<0.05; (D)(E) *P<0.05; **P<0.01. N.S., not significant (*P*>0.05).

Caloric restriction typically extends the lifespan in aging models, including replicative and chronological aging in *S*. *cerevisiae*. The macrolide compound rapamycin mimics the effect of caloric restriction by inhibiting the nutrient-sensitive TORC1 pathway. We examined the possible relationship between pH-mediated extension of chronological lifespan and longevity due to caloric restriction by the reduction of glucose content to 0.5% or the addition of rapamycin. As shown in Fig 5A, caloric restriction and amino acid addition produced beneficial effects on the chronological lifespan. In addition, rapamycin extended the lifespan of cells in both SD and SC media (Fig 5B and 5C), suggesting the effect of amino acids on lifespan extension is independent of the TORC1 pathway. Addition of rapamycin extended the chronological lifespan without any detectable effect on pH in the SD medium in stationary cultures (Fig 5D). However, under caloric restriction, the decrease in pH was slightly repressed (Fig 5E), which may be due to early arrest of cell proliferation under the caloric restriction condition.

### Low pH is less toxic for proliferative cells than for those at the stationary phase

**Fig 6:**
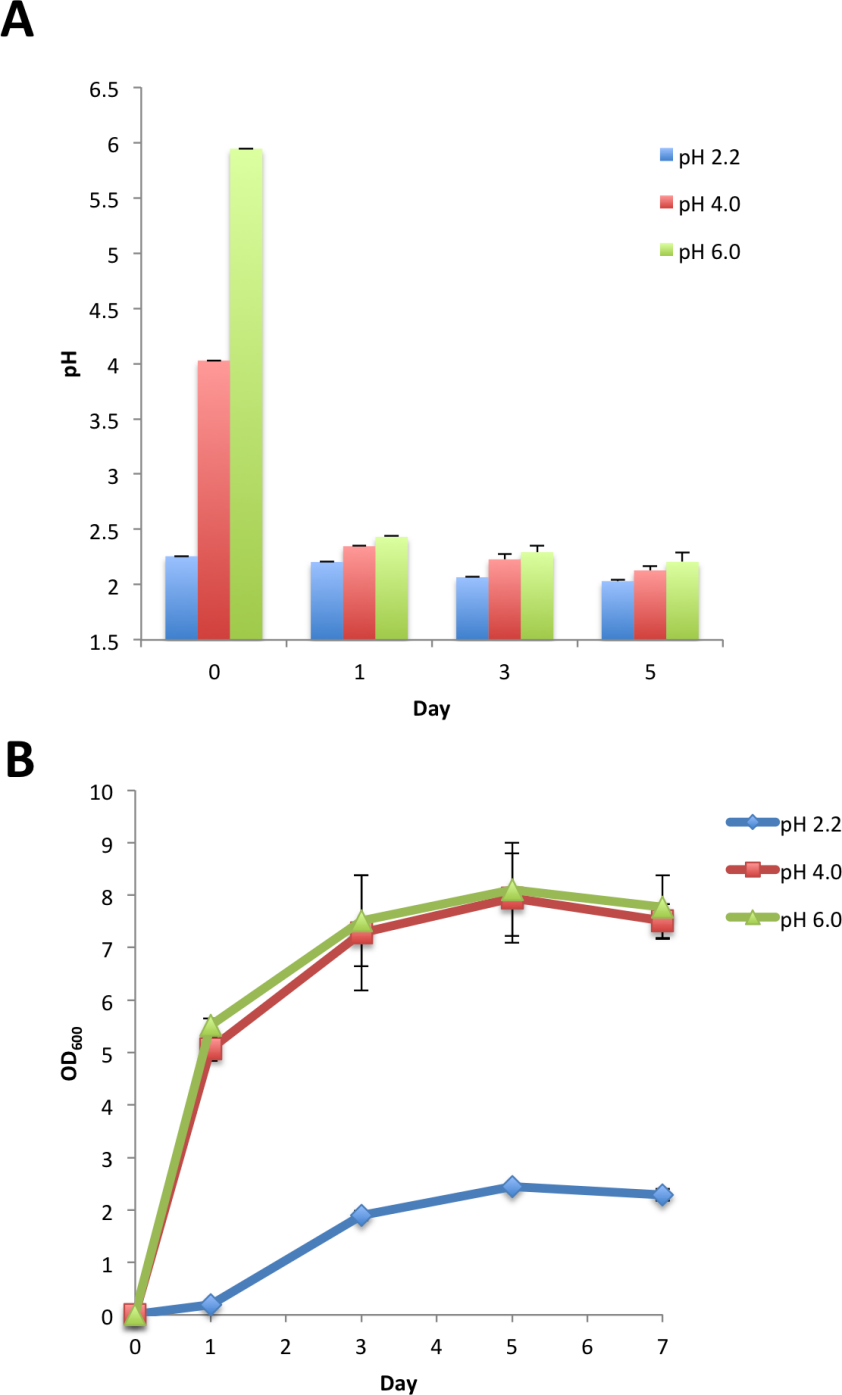
Impact of initial medium pH on final pH and cell growth. YOM36 cells grown in SD medium were transferred to SD medium with pH adjusted to 2.2, 4.0 or 6.0 by NaOH or HCl. Medium pH (A) and cell density (B) of the cultures were measured at the indicated times. Error bars represent the means ± SD (n = 3). (B) *P*<0.01 (pH 2.2 *vs* pH 4.0; pH 2.2 *vs* pH 6.0).

The data presented above suggest a medium pH around 2.5 is a threshold value for maintaining cell viability in yeast cells at the stationary phase. We then tested whether the pH threshold affected cell growth at the exponentially growing phase. Cells precultured overnight in normal SD medium were inoculated into fresh SD medium with an adjusted pH of 2.2, 4.0 or 6.0. As shown in Fig 6, cell growth was impaired, but was not completely suppressed, in SD-pH 2.2 medium. Cell growth did not differ between SD-pH 6.0 and SD-pH 4.0 media. Interestingly, the difference in initial pH values had only a minimal effect on the pH values of the stationary culture. These results suggest the pH threshold value is critical for yeast cells in all phases of growth, but low pH is less toxic for proliferative cells in glucose-rich medium.

### The addition of amino acids suppresses medium acidification and causes chronological longevity in a prototrophic Sigma strain

The strain BY4742, used to construct the prototrophic stain YOM36, is a derivative of strain S288C. To assess the generality of the relationship among amino acids, medium acidification, and longevity, we examined the effect of amino acid addition on the chronological lifespan of Σ1278b, whose genomic divergence from S288C is roughly equal to that between the genomes of two human individuals [23].

**Fig 7:**
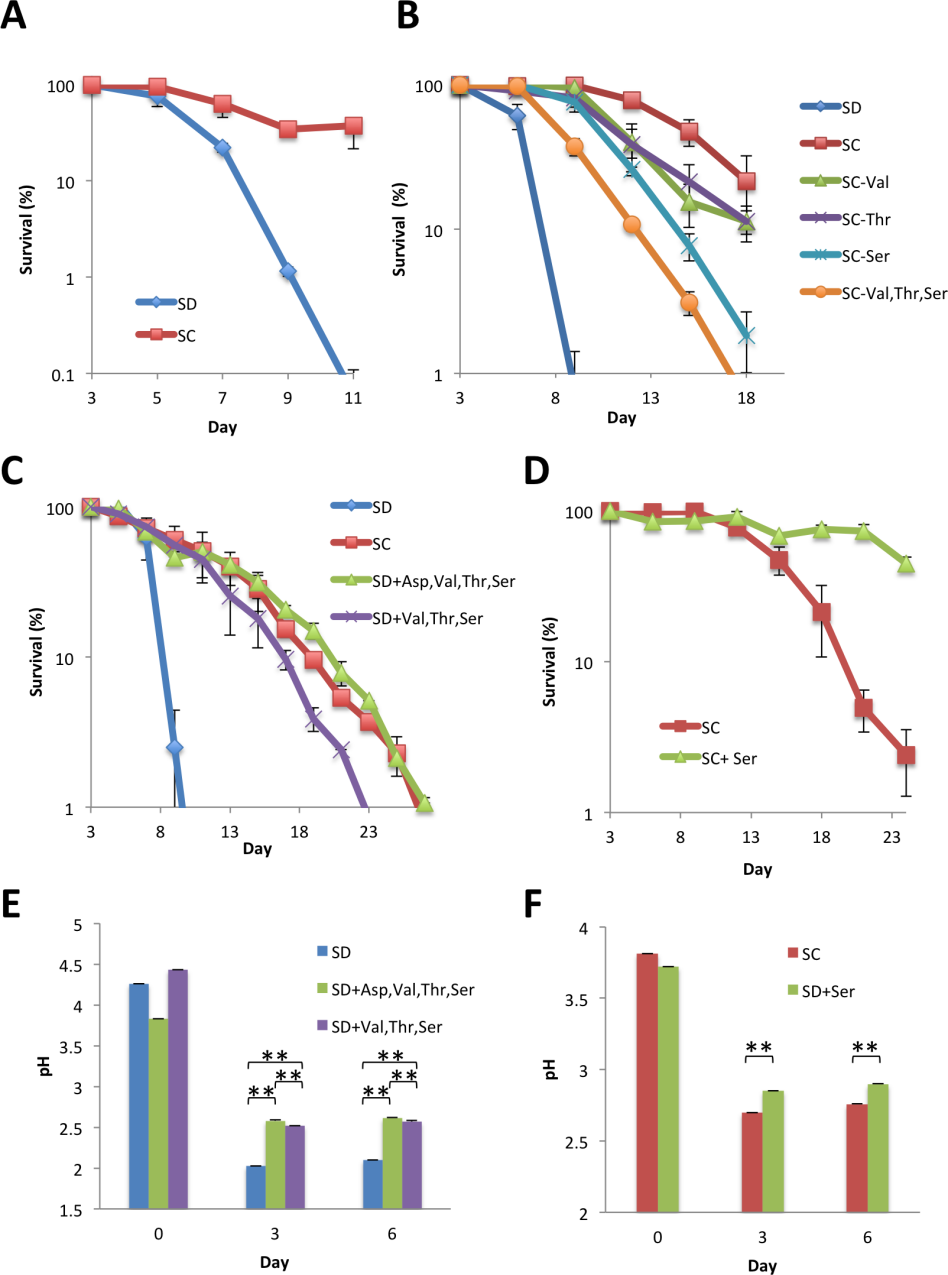
The top four most enriched amino acids are sufficient for amino acid-induced chronological longevity. (A) Effect of non-essential amino acids on the chronological lifespan of Σ1278b. (B) Effect of the elimination of single amino acids from SC medium on chronological lifespan of YOM36. (C) Effect of single or combined addition of top four most enriched amino acids in SC medium. (D) Effect of additional serine. Σ1278b (A) or YOM36 (B-D) cells grown in SD medium were transferred to SD, SC, or media indicated, and CFUs of the cultures were measured at the indicated times. (E) Changes in medium pH of the SD cultures supplemented with top four most enriched amino acids. (F) Changes in medium pH of the SC cultures with additional serine. Error bars represent the means ± SD (n = 3). (A) *P*<0.01; (B) *P*<0.01 (SC *vs* SC-Val, Thr, Ser; SC *vs* SC-Thr), *P*<0.05 (SC vs SC-Val; SC vs SC-Ser); (C) *P*<0.01 (SD+Val, Thr, Ser *vs* SD+Asp, Val, Thr, Ser; SC vs SD+Val, Thr, Ser); (D) *P*<0.01; (E)(F) ** *P*<0.01.

When prototrophic Σ1278b strains were grown on a single nitrogen source, ammonium and asparagine supported cell growth with the shortest doubling time (2.0 h) [24]. Therefore, ammonium was believed to be as the preferred nitrogen source for Σ1278b. We followed previous experiments using this strain, and found that, at day3, the OD_600_ in SD medium was significantly higher than that in SC medium (OD_600_: 6.53±3.2 and 3.61±3.3, respectively; *P*<0.01), and that the medium was more acidified in SD than in SC medium (pH: 2.09±0.01 and 2.70±0.06, respectively; *P*<0.01). The difference in pH between the two media was maintained at day 11 (pH: 1.98±0.01 and 2.49±0.02, respectively; *P*<0.01), indicating that utilization of ammonium as the sole nitrogen source causes hyper-acidification of the medium also in Σ1278b. As shown in Fig 7A, cells in SC medium maintained viability for a longer period than they did in SD, showing that the addition of amino acids impacted the chronological lifespan of Σ1278b. Thus, our results using two divergent *S*. *cerevisiae* strains are suggestive of a conserved relationship among the addition of amino acids, suppression of medium acidification, and chronological longevity.

### The concentration of total amount of amino acids, but not those of specific amino acids, is responsible for the extension of the chronological lifespan of YOM36

Ammonium leads to a shortening of chronological lifespan of an auxotrophic BY4742 under starvation conditions for essential amino acids, which is caused by lack of leucine alone or in combination with histidine [25]. To examine the contribution of specific amino acid(s) to the chronological longevity of the prototrophic strain, we modulated the amino acid composition of SC medium.

SC medium used in this study contains 14 amino acids, each supplemented at a specific final concentration (see Materials and Methods). Among these, serine, threonine, and valine are the top three most enriched amino acids (shown in decreasing order). First, we prepared a series of media with each of these amino acids omitted from SC medium and examined the effect on chronological lifespan. As shown in Fig 7B, removal of threonine or valine slightly reduced the lifespan, and removal of serine alone lead to a further decrease. The effect of the removal of an amino acid appeared to be additive, as simultaneous removal of these three amino acids caused a further decrease in chronological lifespan.

Next, we prepared an SC medium that did not contain any amino acids except for serine, threonine, and valine. As shown in Fig 7C, SD medium with the three amino acids did not completely recapitulate the effect of SC medium on chronological longevity. Further supplementation of aspartic acid (the fourth most enriched amino acid in SC medium) in addition to the three amino acids increased cell survivability comparable to that in SC medium. These results showed that the effect of SC medium on chronological lifespan can be reproduced by adding serine, valine, threonine, and aspartic acid. Moreover, the chronological lifespan was extended further when twice the amount of serine was added to SC medium (Fig 7D), indicating that the amount of amino acids in SC medium is a limiting factor for chronological lifespan. In both cases, the difference in chronological lifespan correlates with that in medium pH at day 3 or day 6 (Fig 7E and 7F), supporting the model that the effect of amino acids on chronological longevity is mediated through the suppression of medium acidification. Interestingly, serine and aspartic acid belong to the preferred class of amino acids for S288C background cells when used as the sole nitrogen source, but threonine belongs to the non-preferred class of amino acids [26].

## Discussion

A previous study showed that concentrations of supplemented amino acids in SC medium affected growth and glucose consumption of auxotrophic yeast cells and concluded that limitation of essential amino acids leads to early growth arrest and short chronological lifespan [13]. Thus, to avoid amino acid shortages in long-term cultures, five-fold higher concentrations of amino acids were developed for supplementation in SC medium in chronological lifespan studies [27, 28]. Although the positive role of essential amino acids in the chronological lifespan of auxotrophic yeast cells has been established, an earlier study showed that non-essential amino acids had negative effects on the replicative lifespan of auxotrophic cells [12]. Rescuing auxotrophic mutations by introducing the corresponding wild-type genes, we constructed a prototrophic yeast cell from a standard laboratory strain and confirmed that supplementation of non-essential amino acids plays a positive role in the chronological lifespan.

In this study we confirmed that supplemented amino acids induce chronological longevity in prototrophic yeast and showed the effect was mediated *via* cell-extrinsic factors. Addition of amino acids improved maximal cell density and suppressed the decrease in medium pH slightly, but significantly. Although relatively resistant to acidic environments, *S*. *cerevisiae* cannot fully grow at a pH below 2.5 [29]. The addition of a pH buffer to the culture medium or the adjustment of the medium to neutral pH by the addition of NaOH extended the chronological lifespan of yeast cells [15, 30]. We consistently showed that modulation of pH by adding a non-metabolized chemical, such as NaOH or HCl, reproduced the effects of amino acid addition on maximal growth or chronological longevity, supporting the model that extracellular H^+^ ion is the cell-extrinsic deleterious factor determining growth limitation and mortality in SD medium. In addition, our results showed that amino acids, even if exhausted at earlier time points, alleviate the acidification of the medium at the stationary phase, contributing to the maintenance of cell viability.

Under standard culture conditions, the medium pH decreases during the culturing of cells and reaches a plateau at the stationary phase. Deleterious effects of medium acidification on yeast viability have been reported [31] and the major cause of medium acidification is assumed to be secreted metabolites, including acetate and other organic acids, produced during fermentative metabolism. A previous study identified acetic acid as a cell-extrinsic mediator of cell death during chronological aging [15]. However, in this study, we observed acetate accumulation in the culture medium occurred only transiently and accumulated acetate was catabolized after day 5. In addition, the maximal levels of acetate concentration were not correlated with the shortness of the chronological lifespan because acetate accumulated more in SC than in SD media cultures. This suggests that acetate is not the sole secreted metabolite that negatively affects the chronological lifespan.

Among the constituents of yeast synthetic medium, glucose was exhausted within 3 days in both SC and SD media cultures. In contrast, the abundance of ammonium was decreased only to 75% of the initial at day 9 in both media, indicating that cells in the stationary culture were under carbon source-depleted, nitrogen source-plentiful conditions. We observed the majority of amino acids in the SC medium disappeared within the first 3 days and concomitantly, the concentration of ammonium ions in SC medium was increased at day 3. Thus, cells preferentially utilized amino acids as a nitrogen source instead of ammonium and excess nitrogen may be released into the medium as ammonium ions before the stationary phase. Excess ammonium within cells causes toxicity in plants and animals and excretion of ammonium has been observed in yeast cells [32]. Moreover, excretion of ammonium ions was found in cells growing as colonies on a solid medium, acting as a signal triggering programmed cell death of aged yeast cells [33, 34]. Thus, excretion of ammonium ions is potentially a mechanism common to aged yeast populations, contributing at least in part to alleviate acidification in aged cultures [33]. We found that the increased ammonium level at day 3 dropped to the original level at day 5 in SC culture, suggesting that medium ammonium was further metabolized during the period. As the medium pH reached plateau both in SD or SC medium after day 3 (Fig 4A; Fig 7E and 7F), utilization of ammonium after the diauxic shift, which proceeded in the absence of glucose, may have a smaller influence on medium acidification than that in the presence of glucose. Ammonium production is a periodical behavior of aged yeast colonies, and ammonium seems to act as a signal causing metabolic changes and growth inhibition in neighboring colonies [33]. Further studies are needed to elucidate the function of excreted ammonium in yeast liquid cultures.

As shown in this study and by others [35], SD medium contains excess ammonium. Yeast cells preferentially utilize nitrogen sources through the catabolite repression mechanism [36, 37]. When multiple nitrogen sources are available, amino acids (except proline) are utilized earlier than are ammonium ions under brewery conditions [38]. In contrast, recent reports have shown that the order of nitrogen source consumption in a synthetic medium mimicking a grape must is slightly different, in that ammonium is utilized earlier than valine, arginine, alanine, tryptophan, and tyrosine [39], and that several genetic variations affect the consumption of nitrogen sources [40]. In this study, we showed that the addition of serine, valine, threonine, and aspartic acid are sufficient for the extension of the chronological lifespan of a BY4742-based prototrophic strain in SD medium (Fig 7). It has been shown that ammonium shortens the chronological lifespan of auxotrophic yeast strains by modulating the nutritional equilibrium between glucose and nitrogen sources [35, 41]. The total amount of added amino acids, rather than their species, may contribute to the altered nutritional equilibrium and thereby chronological longevity. Notably, amino acid synthesis is an energetically costly reaction [42]; thus, utilization of ammonium as the sole nitrogen source may cause a trade-off between growth under nutrient-rich conditions and survival under starvation conditions.

Nitrogenous compounds are incorporated into cells using an active transport system in which plasma membrane H^+^-ATPases are involved. Amino acids are taken up through a general amino acid permease encoded by *GAP1* or through other permeases with a variable degree of specificity for particular sets of amino acids [43]. Ammonium is transported into the cells *via* family proteins Mep1, Mep2 and Mep3 [44]. Uptake of amino acids through Gap1 or other specific permeases is mediated by H^+^-symport systems, driven by a proton motive force generated by the plasma membrane H^+^-ATPase, Pma1 [45, 46]. The mechanism of ammonium uptake remains controversial and electrogenic NH_4_^+^ uniport, NH_3_/H^+^ symport and electroneutral NH_4_^+^/H^+^ antiport were proposed as the mechanisms in plant and mammal orthologs [47]. Ammonium uptake by Mep2, but not by Mep1, showed dependence on the proton gradient through the plasma membrane, suggesting that ammonium transport through Mep2 is facilitated by an inwardly directed proton gradient [48]. In contrast, Mep1 and Mep3 apparently have different transport mechanisms from Mep2, which remain unclear. Biochemical analysis showed that Mep2 acts preferentially at low medium pH and when considering the proposed mechanism that Mep2-dependent ammonium transport does not involve net proton transport, utilization of ammonium rather than amino acids as a nitrogen source may exacerbate the acidification of the medium. Thus, extracellular amino acids are beneficial to yeast cells for two reasons: their catabolized product, ammonium ions, are excreted, contributing to the increase in extracellular pH, and suppressing utilization of ammonium ions whose uptake results in further acidification. The plasma membrane H^+^-ATPase is activated by weak acid stress [49], which is important for the maintenance of intracellular pH under acidic conditions. Lowered expression of *PMA1* encoding H^+^-ATPase rendered cells sensitive to low pH [49]. Interestingly, a fission yeast mutant with longer chronological longevity harbored a mutation in the *pma1*^+^ gene [50].

Previous studies suggested the importance of mitochondrial maintenance for cellular survival during starvation [31]. Since extracellular acidification causes mitochondrial dysfunction, thereby leading to reactive oxygen species-dependent cell death [51], our results provide a path to premature chronological aging caused by differences in available nitrogen sources. In addition, although our results highlight the role of extracellular conditions in the chronological longevity of budding yeast, such an extrinsic effect is not the sole determinant of longevity. Caloric restriction is an intervention that extends chronological longevity by suppressing the nutrient sensor complex TORC1. Acidification of the medium was suppressed under caloric restriction [15, 52] and the addition of amino acids to a caloric medium prevented the decrease in medium pH and increased longevity (Fig 5), consistent with the model that acidification of the medium limits chronological longevity. However, action of the TORC1 inhibitor rapamycin was independent of the medium pH (Fig 5). Moreover, a previous study reported that media exchange between wild-type and *tor1*Δ cultures at the stationary phase had no effect on either strain [5], indicating that cell-intrinsic changes accompanied by a reduction in TORC1 activity mediate chronological longevity.
